# Nerve Stretching in Neuralgia

**Published:** 1877-11

**Authors:** 


### NERVE STRETCHING IN NEURALGIA.

Two cases of nerve stretching for neuralgia are given in the *Revue des Sciences Medicale.* The first recorded was a patient of Paul Vogt's, who had wounded the inner aspect of her right forearm by falling upon a cutting instrument. After the wound had healed, there was much impaired movement of the ring and little fingers, attributed to involvement of the flexor tendons in the cicatrix ; and at the same time the seat of the scar was exquisitely sensitive, especially at one point, which, on being touched, gave rise to acute pain, radiating into the fingers named. Every movement of the hand was painful ; and at first the pain was thought to be due to the presence of a foreign body in the wound. An exploratory incision failed, however, in discovering any such causes for the irritation of the nerve, and the patient's condition remained much the same, until, about a year after, Paul Vogt dissected out the ulnar nerve from the cicatricial tissue surrounding it, and, raising it out of its bed, practiced stretching of the nerve in both directions. The operation was immediately followed by the disappearance of the neuralgia, and in a few weeks the movements of the hand and fingers were re-established. There was no recurrence of the symptoms three years after the operation. The second case is related by Petersen, in which an extensive wound of the upper and inner part of the left leg was followed, on healing, by very severe attacks of deep-seated pain, again suggestive of the presence of some undetected fragment of metal with which the wound was inflicted. In this case also no foreign body was found on cutting down upon the injured limb; but Petersen ascertained that the posterior tibial nerve was very sensitive, and that there was some extravasated blood in its sheath. Stretching was practiced, and with the same fortunate result as in Vogt's case.

